# Role of Neutral Sphingomyelinase-2 (NSM 2) in the Control of T Cell Plasma Membrane Lipid Composition and Cholesterol Homeostasis

**DOI:** 10.3389/fcell.2019.00226

**Published:** 2019-10-15

**Authors:** Charlene Börtlein, Fabian Schumacher, Burkhard Kleuser, Lars Dölken, Elita Avota

**Affiliations:** ^1^Institute for Virology and Immunobiology, University of Würzburg, Würzburg, Germany; ^2^Department of Toxicology, Institute of Nutritional Science, Faculty of Mathematics and Natural Science, University of Potsdam, Nuthetal, Germany; ^3^Department of Molecular Biology, University of Duisburg-Essen, Essen, Germany

**Keywords:** neutral sphingomyelinase-2, T cell receptor, plasma membrane, lyso-phospholipids, diacylglycerol, cholesteryl ester

## Abstract

The activity of neutral sphingomyelinase-2 (NSM2) to catalyze the conversion of sphingomyelin (SM) to ceramide and phosphocholine at the cytosolic leaflet of plasma membrane (PM) is important in T cell receptor (TCR) signaling. We recently identified PKCζ as a major NSM2 downstream effector which regulates microtubular polarization. It remained, however, unclear to what extent NSM2 activity affected overall composition of PM lipids and downstream effector lipids in antigen stimulated T cells. Here, we provide a detailed lipidomics analyses on PM fractions isolated from TCR stimulated wild type and NSM2 deficient (ΔNSM) Jurkat T cells. This revealed that in addition to that of sphingolipids, NSM2 depletion also affected concentrations of many other lipids. In particular, NSM2 ablation resulted in increase of lyso-phosphatidylcholine (LPC) and lyso-phosphatidylethanolamine (LPE) which both govern PM biophysical properties. Crucially, TCR dependent upregulation of the important T cell signaling lipid diacylglycerol (DAG), which is fundamental for activation of conventional and novel PKCs, was abolished in ΔNSM cells. Moreover, NSM2 activity was found to play an important role in PM cholesterol transport to the endoplasmic reticulum (ER) and production of cholesteryl esters (CE) there. Most importantly, CE accumulation was essential to sustain human T cell proliferation. Accordingly, inhibition of CE generating enzymes, the cholesterol acetyltransferases ACAT1/SOAT1 and ACAT2/SOAT2, impaired TCR driven expansion of both CD4^+^ and CD8^+^ T cells. In summary, our study reveals an important role of NSM2 in regulating T cell functions by its multiple effects on PM lipids and cholesterol homeostasis.

## Introduction

Neutral sphingomyelinase-2 (NSM2) is the best studied NSM of the four mammalian sphingomyelinases that are active at neutral pH. It is intensively involved in cellular physiology and pathology (Shamseddine et al., 2015). The NSM2 protein, encoded by the SMPD3 gene, has two N-terminal hydrophobic segments associated with cytosolic membrane leaflets and a C-terminal catalytic site (Hofmann et al., 2000). In addition to phosphorylation (Filosto et al., 2010, 2012), a conformational switch following binding to phosphatidylserine (PS) has been proposed to be crucial in enzymatic activation (Airola et al., 2017; Shanbhogue et al., 2019). NSM2-catalyzed sphingomyelin breakdown commonly occurs in response to cellular stress and regulates bone mineralization. Being most abundant in brain, NSM2 is also ubiquitously expressed including cells of the immune system. The role of NSM2 in cytokine (IL1-β, TNF-α, and IFN-γ) induced inflammation and bacterial infections is well established (Shamseddine et al., 2015; Wu et al., 2018) as is its role in cytotoxic effects of cancer chemotherapeutics. However, the involvement in pathogenesis and perpetuation of human tumors seems to be tumor specific (Bhati et al., 2008; Kim et al., 2008; Henry et al., 2013).

Tonnetti et al. (1999) first reported NSM activation after antibody ligation of the T cell receptor (TCR) already 20 years ago. NSM2-deficient mice show high embryonic lethality, dwarfism and fragile bones. This prevented studies on NSM2 function in the immune system (Stoffel et al., 2005; Alebrahim et al., 2014). Furthermore, the lack of specific and sensitive antibodies for detection of the rather sparse amounts of NSM2 expressed in T cells hampered the progress in the field. Nevertheless, NSM2 proved to be essential for TCR signal amplification and sustainment at low antigen doses inducing PKCζ dependent microtubule polarization and vesicular transport (Bortlein et al., 2018). It was shown that T cell morphological polarization and directional migration in response to chemotactic signals are dependent on intact NSM2 activity (Collenburg et al., 2017). Studies on measles virus contacted T cells and tumor cells revealed a strong impact of sphingomyelinase activity and sphingolipids in general on T cell cytoskeleton dynamics (Zeidan et al., 2008; Gassert et al., 2009; Creekmore et al., 2013).

Neutral sphingomyelinase-2 is palmitoylated and predominantly resides at the inner plasma membrane (PM) leaflet mediating sphingomyelinase-dependent ceramide formation there (Hinkovska-Galcheva et al., 1998; Tani and Hannun, 2007). Alternatively, NSM2 was found in the Golgi compartment of primary mouse chondrocytes where it regulated sphingolipid and diacylglycerol (DAG) homeostasis (Stoffel et al., 2016). There is increasing evidence for the importance of NSM2 function in the generation of ceramides. Overexpression of NSM2 in the breast cancer cell line MCF7 showed 60% upregulation of Cer (Marchesini et al., 2003). Pharmacological NSM2 inhibition resulted in the accumulation of unsaturated long-chain sphingomyelins SM36 and SM38 in the mouse brain (Tan et al., 2018) and ceramides with a fatty acid chain length of 16 to 26 were less abundant in fibroblasts from SMPD3 deletion mutant *fro/fro* mice. Notable, accumulation of cholesterol was also observed in these cells (Qin et al., 2012). A key shortcoming of all previous studies is that they were performed on total cell extracts. Accordingly, they did not allow for assignment of NSM2 activity to cellular compartments or to T cell specific functions. Although NSM2 is now well described to be important for the formation of cholesterol-rich microdomains that promote lipid and protein segregation, the mechanism of how ceramide platforms and specifically NSM2 orchestrate PM structural and signaling properties upon TCR stimulation remain unclear (Eich et al., 2016; Tan et al., 2018). We therefore performed lipidomics of PM fractions isolated from NSM2-deficient and sufficient Jurkat cells to study the NSM2 dependent regulation of sphingolipids and other types of structural and functional PM lipids upon TCR ligation with α-CD3 antibody. NSM2 proved to be primarily active at the PM rather than at the intracellular organelles. Lyso-phospholipids involved in regulation of membrane mechanics and curvature, lyso-phosphatidylcholine (LPC) and lyso-phosphatidyl-ethanolamine (LPE), were upregulated in NSM2-deficient cells. Importantly, the generation of the signaling lipids after TCR ligation, namely diacylglycerols (DAG) was dependent on NSM2 activity. As a result of imbalanced uptake and efflux, cholesterol accumulated in NSM2-deficient cells, which were unable to activate the SREBP2 transcription factor, a master regulator of lipid metabolism. Most strikingly, NSM2 ablation largely prevented accumulation of cholesteryl esters (CE) in response to TCR ligation. At a functional level, prevention of CE generation translated into a loss of sustained T cell activation.

## Materials and Methods

### Ethics Statement

Primary human cells from healthy blood were obtained through the blood donor program of the Department of Transfusion Medicine, University of Würzburg, and analyzed anonymously. All experiments involving human material were conducted according to the principles expressed in the Declaration of Helsinki and ethically approved by the Ethical Committee of the Medical Faculty of the University of Würzburg. Written informed consent from blood donor program participants was not required per ethical approval.

### Jurkat Cell Culture, Transfection, and Starvation Assays

CRISPR/Cas9-edited Jurkat cells deficient for NSM2 (ΔNSM) (Bortlein et al., 2018) cells were cultured in RPMI/10%FBS or in 0%FBS for serum starvation experiments and SREBP2 cleavage analysis, proliferation assays or cell synchronization before α-CD3 mediated TCR stimulation. SREBP2 specific antibody (ab30682, abcam) was used to detect full length and cleaved SREPB2 protein in Western blot of the lysates of CTRL and ΔNSM Jurkat cells after cultivation in medium supplemented or not with serum for 24 h. Cell death was analyzed by life flow cytometry of propidium iodide (Beckton-Dickinson Biosciences, Pharmingen) labeled Jurkat cells done according to manufacturers’ protocol. 1 × 10^6^ Jurkat cells were nucleofected with 5 μg plasmid pcDNA3.1-NSM2-GFP DNA expressing human NSM2-GFP fusion protein (kindly provided by Thomas Rudel) using Nucleofector Technology and program X-001 from Lonza (Basel, Switzerland) followed by live cell imaging.

### Plasma Membrane Isolation and Validation

2 × 10^7^ CTRL and ΔNSM Jurkat cells were starved in RPMI/0.5%FBS for 2 hrs and left unstimulated or stimulated for 10 min with the α-CD3 (clone UCHT-1) crosslinked with the goat α-mouse IgG (both 5 μg/ml). Plasma membranes were isolated by Minute Plasma Membrane Protein Isolation Kit (Invent Biotechnologies, Inc., United Kingdom) according to manufacturers’ protocol. Up to four isolations were pooled for one PM preparation used for lipid analysis. Three preparations were analyzed for each type of cells or stimulations.

Alternatively PMs were isolated as the giant plasma membrane vesicles (GPMVs) as described previously (Sezgin et al., 2012). Shortly, 4 × 10^7^ Jurkat cells were washed twice with GPMV buffer (10 mM HEPES, 150 mM NaCl_2_, mM CaCl_2_, pH7.4) and incubated in 30 ml GPMV buffer containing 2 mM NEM as a vesiculation agent at 37°C for 1 h. Cell supernatant containing GPMVs was centrifuged at 100*g* for 10 min three times to remove cell debris and upper 20 ml was centrifuged at 20,000*g* at 4°C for 1 h. The pellet containing PMs was lysed in 1% TritonX100 containing lysis buffer and 10 μg protein was used for Western blot analysis to estimate purity of PM preparation.

Plasma membrane preparations were validated by Western blot analysis using Lck (clone 3A5, Santa Cruz Biotechnology, Inc.), Actin (Sigma Aldrich, Germany) and AIF (clone D39D2, Cell Signaling Technology) specific antibodies.

### Lipid Extraction for Mass Spectrometry Lipidomics

Mass spectrometry-based lipid analysis was performed by Lipotype GmbH (Dresden, Germany) as described (Sampaio et al., 2011). Lipids were extracted using a two-step chloroform/methanol procedure (Ejsing et al., 2009). Samples were spiked with internal lipid standard mixture containing: cardiolipin 16:1/15:0/15:0/15:0 (CL), ceramide 18:1;2/17:0 (Cer), diacylglycerol 17:0/17:0 (DAG), hexosylceramide 18:1;2/12:0 (HexCer), lyso-phosphatidate 17:0 (LPA), lyso-phosphatidylcholine 12:0 (LPC), lyso-phosphatidylethanolamine 17:1 (LPE), lyso-phosphatidylglycerol 17:1 (LPG), lyso-phosphatidylinositol 17:1 (LPI), lyso-phosphatidylserine 17:1 (LPS), phosphatidate 17:0/17:0 (PA), phosphatidylcholine 17:0/17:0 (PC), phosphatidylethanolamine 17:0/17:0 (PE), phosphatidylglycerol 17:0/17:0 (PG), phosphatidylinositol 16:0/16:0 (PI), phosphatidylserine 17:0/17:0 (PS), cholesterol ester 20:0 (CE), sphingomyelin 18:1;2/12:0;0 (SM) and triacylglycerol 17:0/17:0/17:0 (TAG). After extraction, the organic phase was transferred to an infusion plate and dried in a speed vacuum concentrator. 1st step dry extract was re-suspended in 7.5 mM ammonium acetate in chloroform/methanol/propanol (1:2:4, V:V:V) and 2nd step dry extract in 33% ethanol solution of methylamine in chloroform/methanol (0.003:5:1; V:V:V). All liquid handling steps were performed using Hamilton Robotics STARlet robotic platform with the Anti Droplet Control feature for organic solvents pipetting. Sphingolipids (Cer and SM) were additionally analyzed at the University of Potsdam. Lipids from PM fractions and cell organelles were extracted using methanol/chloroform (2:1, V:V) as described (Gulbins et al., 2018). The extraction solvent contained C17 Cer (18:1;2/17:0) and C16-d_31_ SM (18:1;2/16:0- d_31_) (both Avanti Polar Lipids) as internal standards.

### MS Data Acquisition

Samples were analyzed by direct infusion on a QExactive mass spectrometer (Thermo Scientific) equipped with a TriVersa NanoMate ion source (Advion Biosciences). Samples were analyzed in both positive and negative ion modes with a resolution of R(*m/z* = 200) = 280,000 for MS and R(*m/z* = 200) = 17,500 for MSMS experiments, in a single acquisition. MSMS was triggered by an inclusion list encompassing corresponding MS mass ranges scanned in 1 Da increments (Surma et al., 2015). Both MS and MSMS data were combined to monitor CE, DAG, and TAG ions as ammonium adducts; PC, PC O-, as acetate adducts; and CL, PA, PE, PE O-, PG, PI, and PS as deprotonated anions. MS only was used to monitor LPA, LPE, LPE O-, LPI, and LPS as deprotonated anions; Cer, HexCer, SM, LPC, and LPC O- as acetate adducts. Additional Cer and SM analyses (University of Potsdam) were carried out with a 1260 Infinity LC system coupled to a QTOF 6530 mass spectrometer (Agilent Technologies) operating in the positive electrospray ionization mode (ESI+). The precursor ions of Cer or SM species (differing in their fatty acid chain lengths) were cleaved into the fragment ions *m/z* 264.270 or *m/z* 184.074, respectively (Kachler et al., 2017).

### Data Analysis and Post-processing

Data were analyzed with Lipotype developed lipid identification software based on LipidXplorer (Herzog et al., 2011, 2012). Data post-processing and normalization were performed using an in-house developed data management system. Only lipid identifications with a signal-to-noise ratio >5, and a signal intensity 5-fold higher than in corresponding blank samples were considered for further data analysis. All lipids below amount of 0.5 pmol were removed from further analysis. Also occupation threshold was applied to keep only those lipids that were present at least in two experimental replicates from analyzed control cells.

### Lipid Nomenclature

Lipid species are annotated according to their molecular composition as NAME <sum of the carbon atoms in the hydrocarbon moiety>:<sum of the double bonds in the hydrocarbon moiety>;<sum of hydroxyl groups>. For example, in case of sphingolipids, SM 34:1;2 denotes a sphingomyelin species with a total of 34 carbon atoms, 1 double bond, and 2 hydroxyl groups in the ceramide backbone.

Lipid subspecies annotation contains additional information on the exact identity of their acyl moieties and their sn-position (if available). For example, PC 18:1;0_16:0;0 denotes phosphatidylcholine with octadecenoic (18:1;0) and hexadecanoic (16:0;0) fatty acids, for which the exact position (sn-1 or sn-2) in relation to the glycerol backbone cannot be discriminated (underline “_” separating the acyl chains). PC O- denotes an ether- phosphatidylcholine. Raw data set of lipid measurements in pmol are provided in Supplementary Material as Excel file.

### Detection of Ca^2+^ Mobilization

For Ca^2+^-mobilization experiments, Jurkat cells (1 × 10^6^) were loaded with 1 μM Fluo-4 as cell-permanent acetoxymethyl (AM) ester (Molecular Probes, Invitrogen) in Hanks balanced salt solution (HBS) (without CaCl_2_, MgSO_4_, and phenol red) containing 5% FCS and 25 mM HEPES (pH 7.5) according to manufacturers’ protocol. Ca^2+^-flux was determined over time by flow cytometry after passive ER Ca^2+^ depletion induced by 1 μM thapsigargin (Sigma-Aldrich Germany) in Ca^2+^ free HBS followed by addition of 2 mM Ca^2+^ or after active TCR dependent ER Ca^2+^ release after TCR ligation with α-CD3 antibody (10 μg/ml) (clone UCHT-1; Beckton-Dickinson Biosciences Pharmingen) crosslinked with the goat α-mouse IgG (Dianova, Germany) and added in complete Hanks medium (supplemented with 2 mM CaCl_2_).

### Fluorescence Analysis of α-CD3 Stimulated or NBD-Cholesterol Loaded Jurkat Cells

1 × 10^5^ CTRL and ΔNSM Jurkat cells were pre-incubated with α-CD3 antibody (1 μg/ml) (clone UCHT-1; Beckton-Dickinson Biosciences Pharmingen) for 15 min on ice, subsequently transferred onto 8-well glass bottom μ-slides for immunostaining (Ibidi GmbH, Germany) pre-coated with 25 μg/ml α-mouse IgG (Jackson ImmunoResearch Laboratories, Inc., Dianova) (2 h at 37°C) and stimulated for 10 min at 37°C. Jurkat cell activation was stopped by adding warm 4% PFA (in PBS) for 15 min at room temperature, permeabilized with 0.1% Triton-X100 for 5 min, blocked with 5% BSA and incubated with α-STIM1 antibody (D88E10; Cell Signaling Technology) diluted in 1% BSA/PBS overnight at 4°C. Subsequently, cells were stained with α-rabbit Alexa488-conjugated secondary antibody (Invitrogen) for 2 h at RT. Total Internal Reflection Fluorescence (TIRF) microscopy was performed using a Leica AM TIRF microscope and 100x HCX Plan-Apo oil objective (numerical aperture 1.47, working distance 0.1 mm).

CTRL and ΔNSM Jurkat cells were pre-treated or not with C16-ceramide (10 μM) or avasimibe (10 μM) and subsequently seeded on poly-L-lysin coated chamber slides (LabTekII, Nunc) and incubated in RPMI/0%FBS or RPMI/10%FBS supplemented with 5 μM NBD-cholesterol for 2 h. Cells were washed and fixed by adding warm 4% PFA/PBS for 20 min at RT. NBD-cholesterol was visualized by Confocal Laser Scanning Microscopy (CLSM) imaging performed using a LSM 510 Meta (Zeiss, Germany), equipped with an inverted Axiovert 200 microscope and a 40× or 63× EC Plan-Apo oil objective (numerical aperture 1.3 or 1.4, respectively) and laser lines 488. Image acquisition was performed with Zeiss LSM software 3.2 SP2. NBD-cholesterol fluorescence quantification was performed by flow cytometry using FACS Calibur (Becton Dickinson) and analyzed by FlowJo software (TreeStar).

### Quantification of NBD-Cholesterol Efflux

The cholesterol efflux assay was performed as published previously with minor modifications (Song et al., 2015). To assess cholesterol efflux CTRL and ΔNSM Jurkat cells were incubated in phenol red-free RPMI medium containing 5 μM NBD-cholesterol for 2 h at 37°C. Subsequently cells were washed with PBS three times, incubated for 4 h with 50 μg/ml human HDL (Academy Bio-Medical Company, Inc., Biotrend, Germany) as lipid acceptor, and the medium and cell lysates in 0.1% Triton X-100 were collected. FI in the medium and cells were measured in a black polystyrene 96-well plates in the fluorescence reader at a wavelength of 469 nm for excitation and 537 nm for emission. The efflux was calculated by dividing the fluorescence intensity in the medium by the sum of the whole NBD-cholesterol fluorescence intensity in the medium and cell lysate together.

### Labeling With C16-Ceramide and Cholesterol Quantification

A total of 2.5 × 10^7^ Jurkat or Jurkat-ΔNSM cells were extensively washed and re-suspended in RPMI/2% FBS containing 10 μM ω-azido-C16-ceramide (Collenburg et al., 2016), incubated overnight at 37°C and washed three times with HBSS. 1 × 10^6^ cells were used for click-reaction with 20 μM Click-IT Alexa 488 DIBO Alkyne (Life Technologies) and analyzed by flow cytometry to confirm efficient C16-Cer delivery to the cells. CTRL and ΔNSM Jurkat cells left untreated or loaded with C16-ceramide and total cholesterol was extracted from 1 × 10^6^ cells with 200 μl chloroform-methanol (v/v 2:1) followed by quantification of the total cholesterol and cholesteryl ester by colorimetric assay kit II (BioVision Incorporated, United States) according to manufacturers’ protocol.

### T Cell Isolation, Inhibitor Treatment, and Proliferation Assay

Primary human PBMCs were isolated from peripheral blood obtained from healthy donors by Ficoll gradient centrifugation using Histopaque-1077 (Sigma-Aldrich, Germany). CD4^+^ and CD8^+^ T cells from PBMCs were negatively selected using MagniSort^TM^ Human CD4^+^ or CD8^+^ T Cell Enrichment Kits accordingly (Invitrogen by Thermo Fisher Scientific). Triplets of 1 × 10^5^ T cells were left untreated or treated with 10 μM avasimibe and 2 μg/ml Pyripyropene A, pharmacological inhibitors of cholesterol acyltransferases ACAT1/SOAT1 and ACAT2/SOAT2, respectively (both Sigma-Aldrich, Germany), for 2 h and added α-CD3- (clone UCHT-1) alone or together with CD28-specific antibody (clone CD28.2) (1 μg/ml) (both: Beckton-Dickinson Biosciences Pharmingen) on ice for 20 min, subsequently transferred to 96 well plates pre-coated with 25 μg/ml α-mouse IgG (Dianova) (1 h at 37°C). T cells were stimulated for 72 h including a final 24 h labeling period ([^3^H]-thymidine (Amersham)) and proliferation was analyzed using a microplate scintillation counter. Toxicity of pharmacological inhibitors was tested using AnnexinV Apoptosis Detection Kit (Beckton-Dickinson Biosciences Pharmingen).

### Statistical Analyses

Overall, data shown were acquired in at least three independent experiments. For statistical analyses of data sets, unpaired Student’s *t*-test (^∗^*p* < 0.05, ns: non- significant) was used throughout the manuscript. Bars show standard deviations.

## Results

### NSM2 Activity Is Predominantly Located at the Plasma Membrane in Jurkat Cells

**FIGURE 1:**
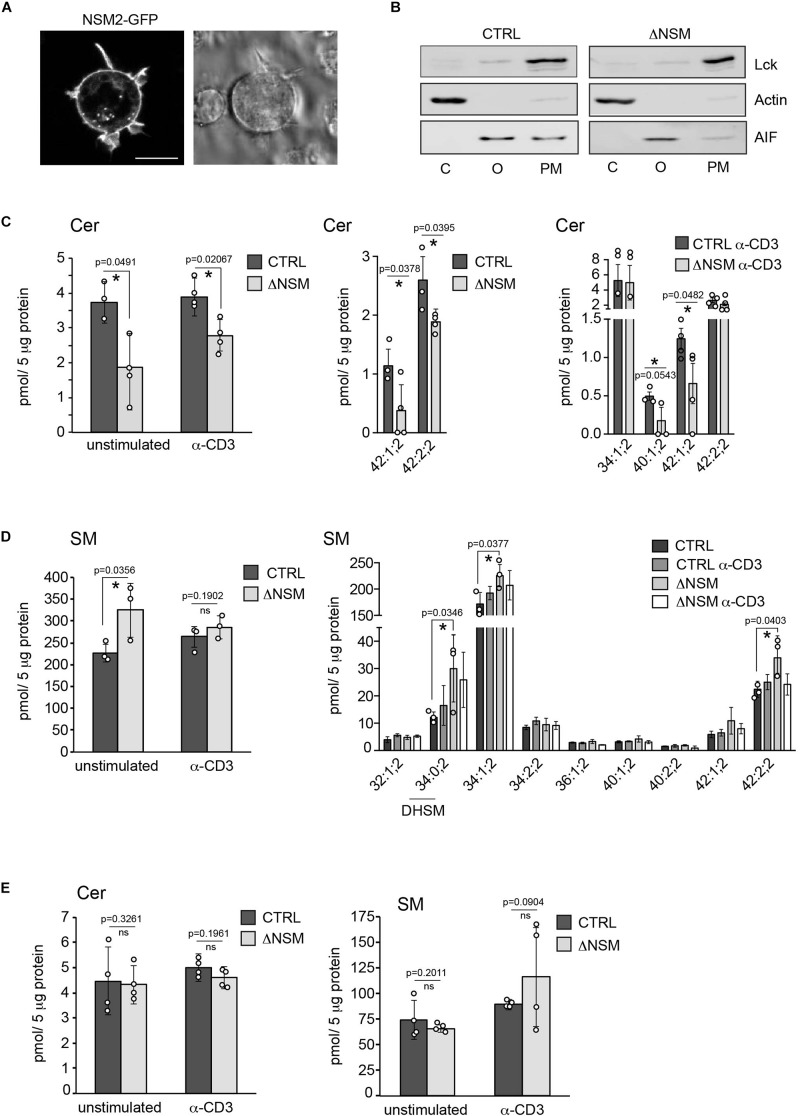
Neutral sphingomyelinase-2 (NSM2) activity is primarily localized at the plasma membrane (PM). **(A)** Jurkat cells were nucleofected with human NSM2-GFP expressing plasmid and images of living cells were taken 24 h after transfection. Left: GFP fluorescence image. Right: differential interference contrast (DIC) image. Scale bar: 10 μM. **(B)** The cell compartment specific localization of actin, Lck and AIF was detected in cytoplasmic (C), organelles (O) and PM fractions of CTRL and ΔNSM Jurkat cells by Western blotting. **(C,D)** Sphingolipid composition of PM fractions of CTRL and ΔNSM cells left unstimulated or α-CD3 stimulated for 10 min. Ceramides [**(C)**, Cer] and sphingomyelins [**(D)**, SM] were analyzed by direct infusion MS/MS or LC-MS/MS. Total levels of 42:1;2 and 42:2;2 Cer species in CTRL and ΔNSM cells detected in both: stimulated and unstimulated cells, by Lipotype and at the University of Potsdam are shown in [**(C)**, left graph]. Distribution of Cer species in unstimulated [**(C)**, middle graph] and α-CD3 stimulated cells [**(C)**, right graph] is shown. Cell or stimulation dependent total SM levels (left graph) and distribution of SM species (right graph) are shown in **(D)**. **(E)** Total Cer (left graph) and SM (right graph) analysis of organelle fractions isolated from CTRL and ΔNSM cells as assessed by LC-MS/MS (*n* = 4). Mean values with standard deviations of the measurements of independently performed fractionations are shown. Each independent fractionation is marked as a circle and *p*-value is shown on the top of significant (marked with asterisks) or not significant differences (ns).

In non-lymphoid cells, NSM2 was found to be associated with the PM, membranes of Golgi and endo-lysosomal compartments. Newly synthesized NSM2 protein was Golgi associated, shuttled to the PM and recycled back through the endosomal system as shown for MCF7 cells (Milhas et al., 2010). To visualize the localization of NSM2, Jurkat cells were transfected with a plasmid encoding NSM2-GFP and subjected to live cell imaging. GFP fluorescence was enriched mainly at the PM, its protrusions and some intracellular vesicles (Figure 1A). To analyze whether subcellular distribution of NSM2 also reflects its activity and regulates PM lipids and their dynamics upon TCR stimulation CRISPR/Cas9-edited Jurkat cells deficient for NSM2 (ΔNSM) were used (Bortlein et al., 2018). Jurkat cells stably transfected with CRISPR/Cas9 plasmid expresing non-specific guide RNA served as control (CTRL). Following 10 min of stimulation by α-CD3, which resulted in robust NSM2 activation (Bortlein et al., 2018), PM fractions were isolated from both CTRL and ΔNSM cells. Isolation efficiency was validated by probing for protein markers specific for cytoplasm, organelles and PM, namely actin, mitochondria-specific apoptosis-inducing factor (AIF) and Src kinase Lck. Faint association of Lck with organelles and the mitochondria-specific protein AIF with PM fractions reflects either contamination with mitochondrial membranes due to the isolation protocol or the formation of the contact sites or junctions between organelles and PM (Figure 1B). We also found mitochondrial proteins in PMs isolated as the giant plasma membrane vesicles (GPMVs) demonstrating objective technical difficulties fully separate organelle and PMs hinting at their close physical communication (Supplementary Figure S1). Mass spectrometry-based lipid analysis of the PM fractions from three independent experiments of unstimulated and α-CD3 stimulated cells was performed by Lipotype GmbH (Dresden, Germany). Diversity of the measured ceramide (Cer) species were generally low, especially in the PM fractions of unstimulated Jurkat cells: only two species in unstimulated and 4 in α-CD3 stimulated cells were detected (Figure 1C, middle and right graphs). Independent measurement of PM Cer was performed in parallel at the University of Potsdam and also here detected ceramide concentrations and species were similar to those measured by Lipotype (Supplementary Figure S2A). Both independently done measurements showed reduced total amounts of ceramide species 42:1;2 and 42:2;2 in ΔNSM cells (Figure 1C, left graph). Hexosyl-ceramide (HexCer) content in Jurkat PM was not affected by NSM2 ablation (Supplementary Figure S2B) indicating that HexCer synthesis is independent of NSM2 regulated sphingolipid metabolic pathways.

Reduction of Cer in the PM of ΔNSM cells correlated with the upregulation of major sphingomyelin (SM) species and total SM in PM by about 30 percent in unstimulated ΔNSM cells (Figure 1D) indicating activity of NSM2 sphingomyelinase at the PM. Measured Cer amounts did not increase in PM after α-CD3 stimulation as could be expected after TCR dependent NSM2 activation (Figure 1C). The result possibly reflects the insufficient sensitivity of methods used here to detect local, TCR signal dependent generation of ceramides after NSM2 activation. Also potential metabolic turnover of ceramides after T cell stimulation cannot be excluded.

Disrupted homeostasis of SM, Cer, and DAG was previously observed in the Golgi compartment of primary chondrocytes from *smpd3^–^/^–^* mouse (Stoffel et al., 2016). Therefore, Cer and SM species were measured in the organelles isolated from CTRL and NSM2 deficient cells (Figure 1E and Supplementary Figure S2C). Surprisingly, we found that cellular organelles did not show significant changes in either steady state or α-CD3 stimulated total levels or subspecies composition of Cer and SM. Data implicate that the majority of enzymatically active NSM2 in Jurkat cells is localized at the PM rich in anionic phospholipid phosphatidylserine (PS), which supports constitutive and TCR stimulated activity of NSM2.

### Enhanced PM Lyso-Phospholipid Content in NSM2 Deficient Jurkat Cells

**FIGURE 2:**
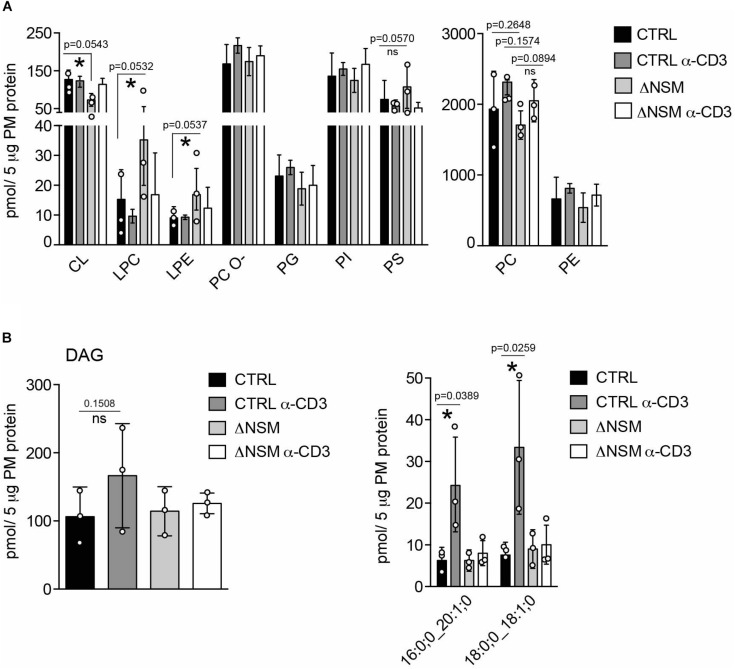
Neutral sphingomyelinase-2 (NSM2) regulates plasma membrane (PM) content of signaling lipids: lyso-glycerophospholipids (LP) and diacylglycerols (DAG). CTRL or ΔNSM cells were left unstimulated or α-CD3 stimulated for 10 min and total amounts of major glycerophospholipids (GPL) **(A)**: cardiolipin CL, lyso-phosphatidylcholine LPC, lyso-phosphatidylethanolamine LPE, ether-phosphatidyl-choline PC-O, phosphatidylglycerol PG, phosphatidylinositol PI, phosphatidylserine PS) and total diacylglycerol (DAG) [**(B)**, left graph] or α-CD3 regulated DAG species [**(B)**, right graph] were quantified by direct infusion MS/MS. Mean values with standard deviations of the measurements of three independently performed fractionations are shown. Each independent fractionation is marked as a circle and *p*-value is shown on the top of significant (marked with asterisks) or not significant differences (ns).

To evaluate whether NSM2 deficiency also affected the accumulation of PM lipids other than sphingomyelin species, we analyzed 14 glycerophospholipid (GPL) types (including four plasmalogens containing a vinyl-ether bond). The most abundant species (higher than 10 pmol per sample) included two major structural lipids, phosphatidylcholines (PC) and phosphatidylethanolamines (PE), which were slightly but not significantly upregulated upon α-CD3 stimulation (Figure 2A, right graph). More detailed analysis within the group of most expressed PC species (more as 100 pmol per sample) showed a significant reduction only for two PC species in α-CD3 stimulated ΔNSM cells (Supplementary Figure S3A), indicating that the total content of the major structural lipids is regulated in an NSM2-independent manner.

Although typically localized to the mitochondrial inner membrane, the glycerophospholipids cardiolipins (CL) and its building blocks phosphatidylglycerols (PG) were also detected in the PM fractions. In the PM of unstimulated ΔNSM cells, CL was significantly less abundant, while no significant differences for PG were observed (Figure 2A, left graph; Supplementary Figure S3B). It remains to be clarified whether CL and PG truly are associated with PM or rather localized to organelle/PM contact sites reflecting NSM2-dependent regulation of CL in mitochondrial membranes.

Lyso-phosphatidylcholine (LPC) and lyso-phosphatidylethanolamine (LPE) are bioactive molecules that can modify cell membrane mechanical properties and curvature (Shindou et al., 2009). Interestingly both, LPC and LPE, were more abundant in unstimulated ΔNSM cells (Figure 2A, left graph; Supplementary Figure S3C) and thus represented the major glycerophospholipid species which are affected by basal NSM2 activity.

### TCR-Dependent NSM2 Activation Is Crucial for Diacylglycerol (DAG) Production

DAG accumulating in PM microdomains regulates classic, novel and atypical PKCs important in T cell signaling (Fu et al., 2010; He et al., 2011). Two metabolic pathways produce DAG at the PM. One is mediated by sphingomyelin synthase 2 (SMS2), which transfers the phosphocholine head group from phosphatidylcholine to Cer to produce DAG and SM. Therefore SMS2 can regulate SM, Cer, PC and DAG simultaneously (Gault et al., 2010). Another pathway is governed by phosphatidylinositol-specific phospholipase-Cγ1 (PLCγ1), which hydrolyzes PI (4,5)P_2_ into inositol triphosphates (IP_3_), thereby mobilizing Ca^2+^ and DAG upon TCR activation. NSM2 deficiency did not affect steady state PM DAG production in unstimulated Jurkat cells (Figure 2B). Remarkably, after α-CD3 stimulation, two out of 13 analyzed DAG species were significantly upregulated in NSM2-expressing but not in NSM2-deficient cells (Figure 2B, right graph; Supplementary Figure S4). Interestingly, TCR signaling specifically induced the production of more saturated DAG species that contain only one double bound (Supplementary Figure S4). Our data indicate that NSM2 is required for TCR signal dependent DAG production.

### NSM2 Activity Is Required for Cholesteryl Ester (CE) Production in Jurkat Cells

**FIGURE 3:**
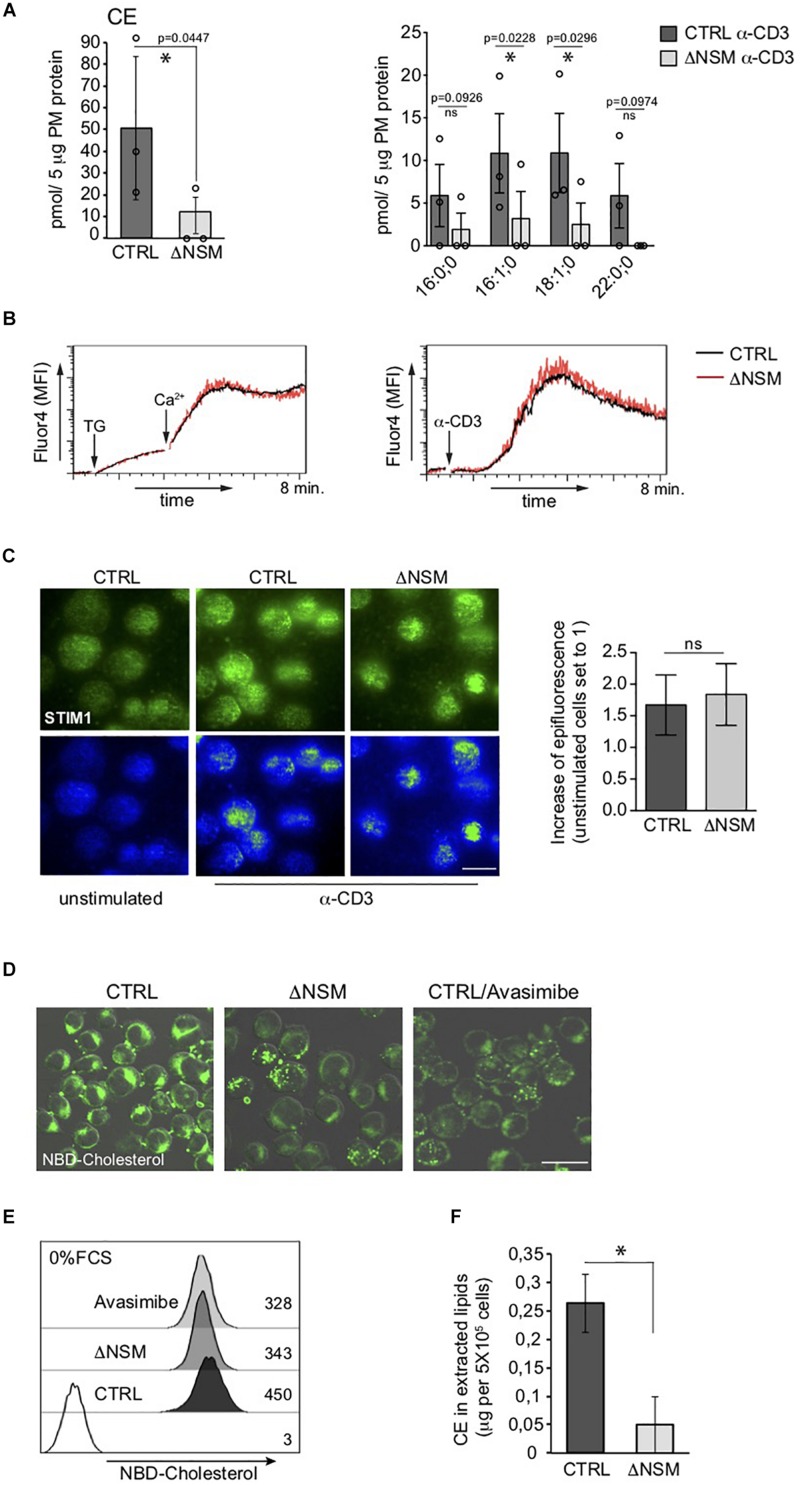
Unimpaired ER-plasma membrane (PM) junction formation and lack of cholesteryl ester (CE) production in TCR stimulated neutral sphingomyelinase-2 (NSM2) deficient T cells. **(A)** Total CE (left graph) and the distribution within CE species (right graph) were measured and are shown in CTRL and ΔNSM cells after α-CD3 stimulation for 10 min. No CE 22:0;0 was detected in ΔNSM cells. Each independent fractionation is marked as a circle and *p*-value is shown on the top of significant (marked with asterisks) or not significant differences (ns). **(B)** Calcium uptake was analyzed in Ca^2+^ sensor Fluo-4 loaded CTRL and ΔNSM cells after ER Ca^2+^ release induced by thapsigargin treatment (left graph) or after α-CD3 stimulation (right graph). **(C)** Representative fluorescence pictures (left panels) and quantification (right graph) of STIM1 epifluorescence measured by total internal reflection fluorescence (TIRF) microscopy are shown for CTRL and ΔNSM cells left unstimulated or α-CD3 stimulated for 10 min on antibody coated surface, fixed, permeabilized and stained for STIM1. Scale bar: 10 μM. **(D,E)** NBD-cholesterol fluorescence was analyzed by confocal microscopy (fluorescence and DIC picture overlays, scale bar: 20 μM; **(D)** and flow cytometry **(E)** in CTRL and ΔNSM cells untreated or 2 h pre-incubated with 5 μM avasimibe and loaded with 5 μM NBD-cholesterol for additional 2 h in cell culture medium without serum. Mean fluorescence intensity (MFI) values are shown in **(E)**. **(F)** CE was detected in total lipids extracted from CTRL and ΔNSM cells by colorimetric assay. Mean values with standard deviations of the measurements of independent cell extracts are shown (*n* = 3). Significantly reduced CE is marked with asterisk (^∗^*p* < 0.05).

The most striking NSM2-related difference seen in our PM lipidomic analyses concerned all measured cholesteryl ester (CE) species, which accumulated in NSM2-sufficient and barely in NSM2 deficient cells after TCR ligation (Figure 3A). CE is generated after free PM cholesterol transport to the endoplasmic reticulum (ER) by two ER-resident cholesterol acyltransferases ACAT1/SOAT1 and ACAT2/SOAT2. Lipidomic analysis detected CE in PM fractions most likely due to the accumulation of free cholesterol after TCR-dependent sphingomyelinase activation and enhanced ER-PM junction formation which were partially present in purified PM fractions. Analysis of CE in intracellular organelle fraction showed a tendency of reduced CE also there (Supplementary Figure S5). We wanted to clarify whether lack of CE accumulation in ΔNSM cells reflected low CE synthesizing enzyme activity or lack of ER-PM contact formation after TCR engagement necessary for cholesterol transport to ER. For that Ca^2+^ mobilization as crucial for ER-PM tethering (van Vliet et al., 2017) and PM redistribution of the ER Ca^2+^ sensor STIM1 were measured. Thapsigargin-dependent ER calcium release and extracellular Ca^2+^ uptake were NSM2-independent (Figure 3B, left graph). This also applied to TCR- regulated store operated Ca^2+^ entry (SOCE) (Figure 3B, right graph). As revealed by total internal reflection fluorescence (TIRF) microscopy, STIM1 transport toward α-CD3 coated stimulatory surface did occur at equal efficiency in CTRL and ΔNSM cells indicating that formation of PM-ER contact sites was unaffected (Figure 3C).

Next, we wanted to know if CE production is generally impaired in NSM2-deficient cells prior to TCR stimulation. For that we used an assay that indirectly measures CE production rate as the increase in relative NBD fluorescence of loaded NBD-cholesterol after absorbance of it in PM and transport from polar double leaflet membranes to non-polar core of cytoplasmic lipid droplets in NBD-cholesterol loaded cells (Lada et al., 2004). The excess of loaded free NBD-cholesterol in PM is removed to ER where NBD-CE is generated and distributed to intracellular compartments (Sparrow et al., 1999). To exclude that high and low density lipoproteins (HDL, LDL) mediated cholesterol uptake from serum present in our cell culture medium, the cells were extensively washed and incubated with NBD-cholesterol in serum-free medium prior to microscopy (Figure 3D) and flow cytometry (Figure 3E). ACAT1/SOAT1 inhibitor avasimibe was included to reduce intracellular CE concentration. ΔNSM and avasimibe-treated CTRL cells showed a comparable decrease in NBD-fluorescence intensity indicative of reduced production of CE also in unstimulated NSM2-deficient cells. To find out if extracellular supply of free cholesterol (provided by serum) can restore CE production in NSM2-deficient cells, CE amounts were determined in lipids extracted from the CTRL or ΔNSM cells cultivated in serum containing medium using colorimetric assay (Figure 3F). Because CE amount was strongly reduced also in ΔNSM cells cultivated in serum-containing medium, CE production is obviously generally impaired in the absence of NSM2.

### Enhanced Cholesterol Accumulation in NSM2-Deficient Cells

**FIGURE 4:**
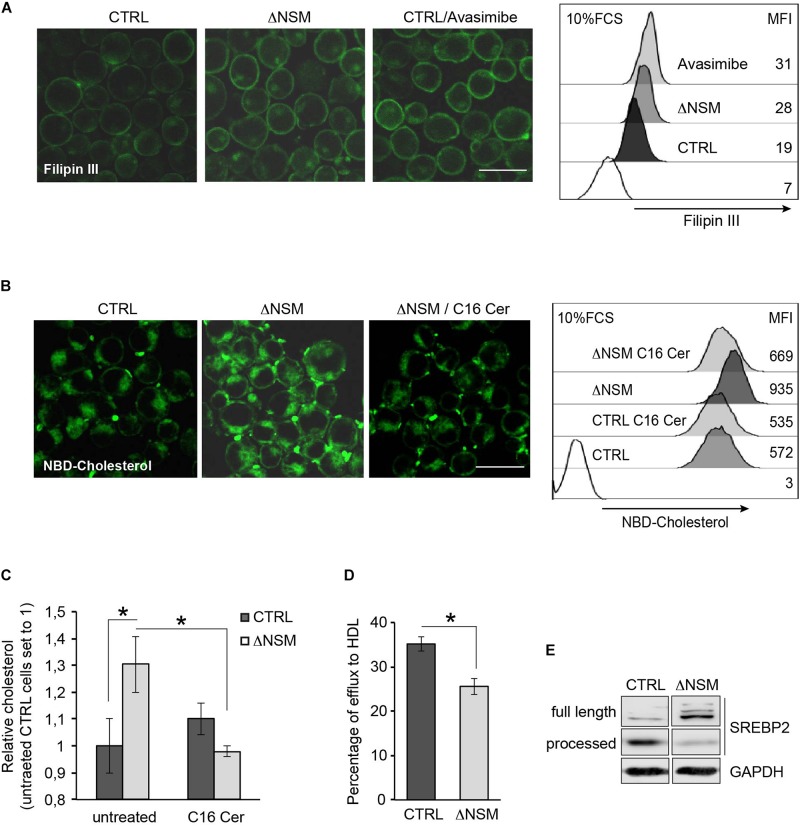
Plasma membrane (PM) and intracellular cholesterol accumulation is regulated by neutral sphingomyelinase-2 (NSM2) dependent ceramide metabolism. **(A)** Cholesterol accumulation in polar cellular membranes was analyzed by confocal microscopy (left panels; overlays of fluorescence and DIC pictures, scale bar: 20 μM) and flow cytometry (right graph) of CTRL and ΔNSM cells left untreated or pre-incubated with 5 μM avasimibe for 2 h, fixed and stained with filipin III. Values of mean fluorescence intensity (MFI) in unstained and filipin III stained cells are shown in the right graph. White histogram in flow cytometry graph shows cells which were left unstained with filipin III. **(B)** NBD-cholesterol uptake was analyzed in CTRL and ΔNSM cells left untreated or loaded overnight with 25 μM C16 ceramide and subsequently loaded with 5 μM NBD-cholesterol for 2 h. Confocal microscopy fluorescence-DIC picture overlays (left panels; scale bar: 20 μM) and flow cytometry measurements (MFI; right panel) are shown. White histogram in flow cytometry graph shows cells which were left unloaded with NBD-cholesterol. **(C)** Cholesterol was detected in CTRL and ΔNSM cells treated as in **(B)** by colorimetric assay. Measurements were normalized against cholesterol amount in untreated CTRL cells set to 1 (*n* = 3). **(D)** Fluorometric analysis of cholesterol efflux was performed in supernatants of NBD-cholesterol loaded cells and cultivated in cell culture medium supplemented with 50 μg/ml human high density lipoprotein (HDL) for 4 h. Efflux was estimated as a percentage of NBD-cholesterol fluorescence in supernatants from total fluorescence in cell lysates and supernatants taken together (*n* = 3). Significant differences are marked with asterisks (^∗^*p* < 0.05). **(E)** Western blot analysis of SREBP2 protein cleavage in CTRL and ΔNSM cells. Cell cultivation was performed in serum containing cell medium.

Conditional knock-out of Acat1/Soat1 in murine T cells impaired CE synthesis and markedly increased both whole and plasma cholesterol levels in mouse T cells (Yang et al., 2016). To determine whether NSM2 deficiency would have a similar effect on free cholesterol levels, we compared cholesterol-specific filipin III fluorescence in ΔNSM and CTRL cells exposed to the ACAT1/SOAT1 pharmacological inhibitor avasimibe. Filipin III showed predominant PM labeling in both, CTRL and ΔNSM cells, with fluorescence intensities in both ΔNSM and avasimibe-treated cells exceeding those observed in CTRL cells (Figure 4A, IF pictures). In quantitative terms, this was confirmed by flow cytometry (Figure 4A, right graph).

In addition to reduced CE production and cholesterol turnover, higher cholesterol uptake rate can cause accumulation of cellular cholesterol in ΔNSM cells. To analyze whether NSM2 and/or ceramides participate in cholesterol uptake in Jurkat cells, we pretreated CTRL and ΔNSM cells with C16-ceramide overnight and loaded with NBD-cholesterol for the last 2 h in the presence of serum as the source of cholesterol carrying lipoproteins LDLs and HDLs. Microscopic and flow cytometry analysis of NBD-fluorescence revealed elevated cholesterol levels in NSM2-deficient cells which were normalized to those in CTRL cells after incubation with C16-ceramide (Figure 4B). Results were confirmed using colorimetric assay detecting total cholesterol (Figure 4C).

To assess the contribution of NSM2 in cholesterol efflux, the supernatants of NBD-cholesterol loaded CTRL and ΔNSM cells were analyzed using human HDL as the lipid acceptor. The fluorescence assessment in cell supernatants showed 10% less efflux in NSM2-deficient cells (Figure 4D). Combined, these data revealed that enhanced uptake and decreased efflux of cholesterol contributes to misbalanced cholesterol homeostasis in ΔNSM cells.

### NSM2-Deficient Cells Do Not Activate SREBP and Undergo Cell Death After Serum Deprivation

Lipid homeostasis is regulated by ER-membrane associated transcription factors, the so-called sterol regulatory element-binding proteins (SREBPs) with SREBP2 being most important in cholesterol biosynthesis pathway (Horton et al., 2002). The activation is tightly controlled by the sterol-sensing SREBP cleavage activating protein (SCAP). At low sterol concentrations SCAP chaperons SREBP protein to the Golgi, where SREBP inactive precursor is cleaved by proteases to initiate the transport to the nucleus. After activating its target genes SREBP increases sterol levels again. So SREBP activation is regulated by sterol concentration dependent feedback loop. We therefore aimed at defining whether enhanced cholesterol levels found in ΔNSM cells deregulated SREBP activity. For that, we analyzed SREBP2 active fragment in cell lysates of NSM2-sufficient and deficient Jurkat cells (Figure 4E). Consistent with the higher cholesterol levels found in ΔNSM cells, inactive full length SREBP2 protein was predominantly expressed in those cells, indicating reduced proteolytic cleavage and activation.

**FIGURE 5:**
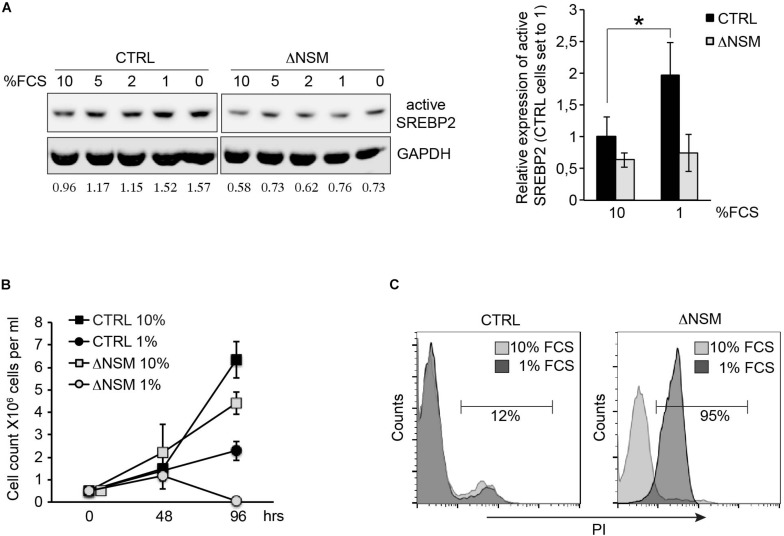
Neutral sphingomyelinase-2 (NSM2) deficiency induced cell death after serum deprivation correlates with lower SREBP2 activation levels. **(A)** Western blot analysis of active SREBP2 expression in CTRL and ΔNSM cells after overnight incubation in cell culture medium containing decreasing amounts of serum. Relative densitometry units for SREBP2 bands are shown below the Western blots. Right graph shows the summary of three independent experiments. Significantly different amount of active SREBP2 in CTRL cells is marked with asterisk (^∗^*p* < 0.05). **(B)** Proliferation was estimated by cell counting of cells cultivated in cell culture medium with normal (10 percent) and low (1 percent) serum content for 4 days (*n* = 3). **(C)** Cell death analysis performed by flow cytometry of propidium iodide (PI) staining of living cells after 4 days of cultivation in cell culture medium containing 10 or 1 percent serum.

To analyze whether NSM2-deficient cells can activate SREBP, we compared SREBP2 cleavage activation in response to the absence of lipid nutrients in CTRL and ΔNSM cells after culture in decreasing serum concentrations for 24 h. NSM2- sufficient, but not NSM2-deficient Jurkat cells gradually increased expression of cleaved SREBP under serum starvation indicating that NSM2 is required to report the absence of lipid nutrients (Figure 5A). We followed the proliferation of cells cultivated in serum supplemented medium and medium without serum for 4 days. As expected, serum absence in medium slowed down CTRL cell proliferation (Figure 5B). In contrast, ΔNSM cells stopped to proliferate completely and underwent massive cell death measured by membrane permeability for propidium iodide at day four after serum deprivation (Figures 5B,C). Thus, these results demonstrated that NSM2 is important to fulfill demands of proliferating Jurkat cells to increase fatty acid biosynthesis necessary for membrane biogenesis particularly in a nutrient poor environment.

### CE Production Is Important for TCR Dependent Primary Human T Cell Proliferation

**FIGURE 6:**
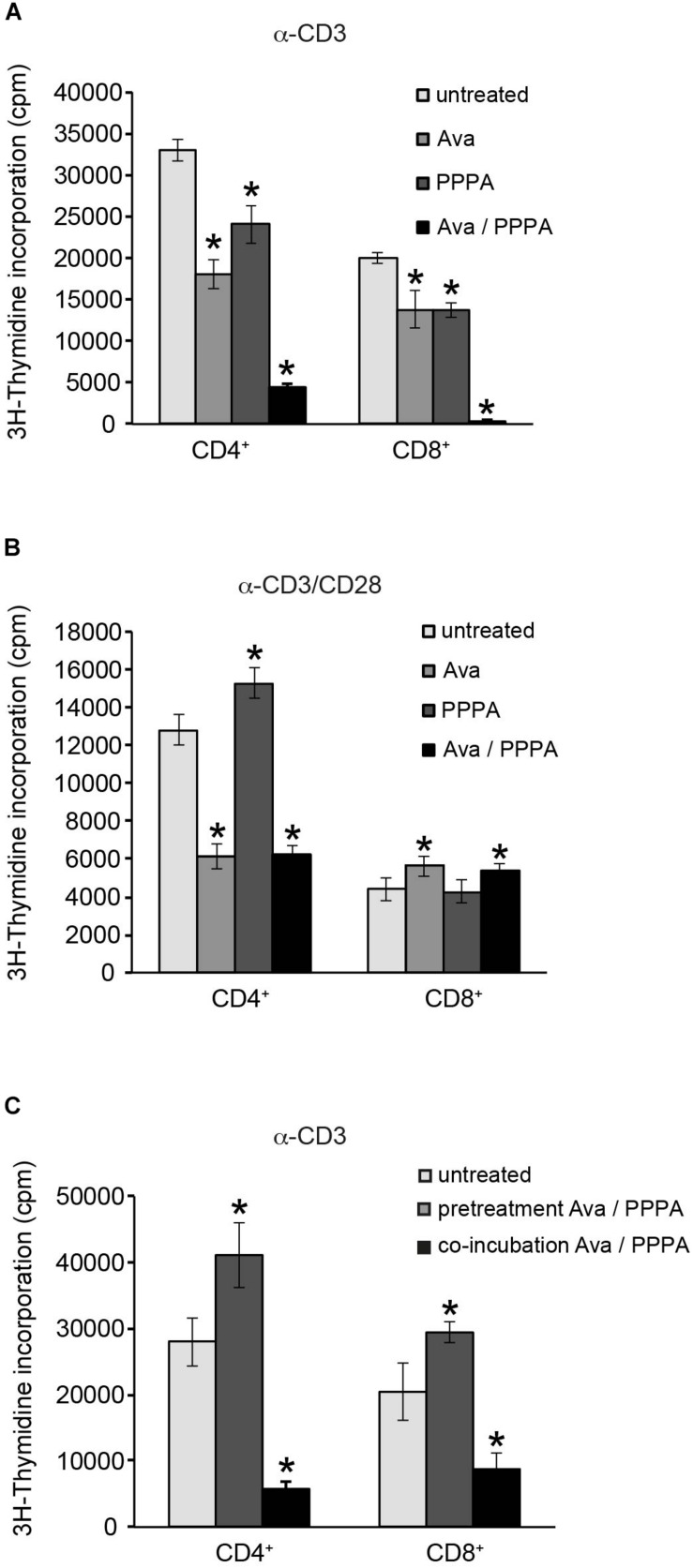
Cholesterol ester synthesis is crucial for sustained α-CD3/α-CD28 driven human CD4^+^ cell proliferation. Purified primary CD4^+^ and CD8^+^ T cells were left untreated or treated with 10 μM ACAT1/SOAT1 specific inhibitor avasimibe (Ava) or ACAT2/SOAT2 specific inhibitor pyripyropene A (PPPA) separately or in combination. Cells were stimulated with α-CD3 alone **(A,C)** or in combination with α-CD28 **(B)** for 3 days. Inhibitors were kept in cell culture medium through the whole experiment **(A,B)** or washed away after 2 h pre-incubation of T cells [**(C)**, pretreatment]. ^3^H-Thymidine was added to the cells after 2 days of stimulation and radioactivity incorporation in cellular DNA was measured after overnight incubation. Mean values with standard deviations of three independent experiments are shown (*n* = 3). Significant differences are marked with asterisks (^∗^*p* < 0.05).

Yang et al. (2016) demonstrated that ACAT1/SOAT1 pharmacological inhibition or T-cell specifc depletion of cholesterol acyltransferase 1 gene significantly potentiated TCR signaling, proliferation and effector functions of mouse CD8^+^ T cells. We tested the two selective pharmacological inhibitors avasimibe (Ava) and pyripyropene A (PPPA), which are specific for ACAT1/SOAT1 and ACAT2/SOAT2, respectively, in proliferation of primary human CD4^+^ and CD8^+^ T cells isolated from peripheral blood of healthy donors. When applied seperately, both ACAT/SOAT inhibitors had significant negative effect on TCR ligation induced proliferation of both CD4^+^ and CD8^+^ T cells (Figure 6A). Apparently, ACAT1/SOAT1 and ACAT2/SOAT2 could partially compensate each other for function as the simultaneous treatment with both inhibitors almost completely abolished the proliferation of T cells. The inhibitor concentrations used for T cell treatment were not toxic upon incubation times used in proliferation assays (Supplementary Figure S6). Co-stimulation of T cells with α-CD3 and α-CD28 antibodies rendered their proliferative responses less sensitive to the inhibitor treatment. However, CD4^+^ T cells, but not CD8^+^ T cells, were sensitive to ACAT1/SOAT1 inhibitor which significantly reduced T cell proliferation alone or in combination with ACAT2/SOAT2 inhibitor (Figure 6B). CD3/CD28 co-stimulated CD8^+^ T cells generally were more resistent to ACAT/SOAT inhibition and showed almost no effect on proliferation with the exception of a relatively minor enhancement of proliferation after avasimibe treatment (Figure 6B). Published data (Yang et al., 2016) and our filipin stainings of PM in avasimibe treated Jurkat cells suggest that higher cholesterol levels in PM may support more efficient TCR clustering and signaling. To dissect whether ACAT/SOAT activity would differentially affect early or late T cell activation, inhibitors were retained in the cultures for 2 h or the entire cell expansion time after stimulation. Short time inhibitor pretreatment of CD4^+^ and CD8^+^ cells had a slightly positive effect on T cell proliferation (Figure 6C) indicating that enhanced PM cholesterol levels may be tolerated or can even support initial TCR signaling. In contrast, retention of ACAT/SOAT inhibition drastically reduced T cells expansion suggesting that extended inhibition of CE synthesis and storage might negatively affect fatty acid homeostasis important for cell proliferation.

## Discussion

Our manuscript describes the first analysis of the NSM2 contribution to the general and TCR dependent lipid regulation at the PM early after stimulation. In line with previous observations for other cell types, the absence of NSM2 affects the levels of ceramides and sphingomyelins. However, major alterations in NSM2- deficient cells were observed in PM but not in organelle fractions of unstimulated and α-CD3 stimulated Jurkat cells indicating that the enzyme may be active in both resting and TCR stimulated cells (Figures 1C–E and Supplementary Figures S2A,C). This does not exclude NSM2 activity in the membranes of intracellular organelles in other cell types or after other modes of activation.

Rouquette-Jazdanian and others (Rouquette-Jazdanian et al., 2005) have shown neutral sphingomyelinase activation and a 46% reduction of SM in cholera toxin B-subunit treated Jurkat cells proposing that NSM should have access also to the major pool of SM in outer PM leaflet by unknown mechanism. Biochemical methods including labeling of the cytosolic face of PM with SM-specific toxin lysenin in electron microscopy (EM) demonstrated that 10–25% of sphingomyelin could be found in the inner leaflet (Murate et al., 2015). Our data show a 30% increase of PM SM in ΔNSM Jurkat cells accompanied with a 30–50% decrease of Cer amounts (Figures 1C,D) specifically in PM, but not in the organelles containing fractions (Figure 1E), indicating that NSM2 is primarily active at PM and have an access only to the SM in the inner PM leaflet. Many stimuli, such as TNF-α, cell confluence and PMA, have been demonstrated to induce NSM2 PM translocation and increase in enzyme activity, indicating together with our data that PM translocation is important for the enzymatic activation of NSM2 (Airola and Hannun, 2013). Although we and others have shown neutral sphingomyelinase activation early after TCR stimulation (Tonnetti et al., 1999; Bortlein et al., 2018), we were not able to show increase of ceramide or decrease of SM levels upon 10 min of TCR ligation using α-CD3 antibody (Figures 1C,D). The data implicate insufficient sensitivity of techniques used in our lipid analysis that allowed us to measure only long term regulation of basal Cer and SM levels at PM in ΔNSM cells, but not short, transient and TCR localized Cer changes upon NSM2 activation in CTRL cells.

To analyze the dynamics of PM lipids upon TCR stimulation is a challenge. Nevertheless, methods of PM isolation and lipid detection are increasingly contributing to the steady data accumulation. Zech et al. analyzed lipids in Jurkat cell PM domains directly engaged to stimulatory α-CD3 coated beads (Zech et al., 2009), revealing raft lipid accumulation in PM regions directly engaged in TCR signaling. Here we first analyze NSM2 contribution to the general TCR-dependent lipid regulation at the whole PM early after TCR stimulation. Majority of analyzed glycerophospholipids were regulated independently of TCR-stimulation (Figure 2A, left graph). Our analysis revealed significantly enhanced basal levels of lysophospholipids LPC and LPE in NSM2-deficient cells (Figure 2A and Supplementary Figure S3C), suggesting a possible molecular mechanism how NSM2 may regulate membrane curvature and mechanic properties in unstimulated T cells (Menck et al., 2017). Interestingly, α-CD3 stimulation normalized the levels of LPC and LPE in ΔNSM cells but still there was a tendency for higher levels of those lipids. However, data evaluation and studies regarding mechanism of how NSM2 regulate lysophospholipid expression stay beyond the scope of this study.

Interestingly, we observed TCR stimulation-specific and NSM2-dependent increase in more saturated DAG species (Figure 2B and Supplementary Figure S4). In NSM2-deficient cells a lack of TCR induced upregulation of DAG correlated with the low activation levels of novel and atypical PKCs and low Ca^2+^ mobilization at suboptimal TCR stimulation conditions (Bortlein et al., 2018).

The most striking observation was CE accumulation in PM fractions of TCR- stimulated cells which was strongly dependent on NSM2 activity (Figure 3A). Enzyme deficient cells had nearly no CE induced after TCR ligation. Several biochemical studies have described sphingomyelinase and PKC-regulated cholesterol esterification in different cell lines (Stein et al., 1992; Lange et al., 2002). Sphingomyelin has the highest affinity to cholesterol in PM. The amount of SM-bound cholesterol was estimated about 35 mole percent of PM cholesterol by using cholesterol binding mutant form of bacterial Perfringolysin O (Das et al., 2014). Sphingomyelinase activity is believed to be required for cholesterol mobilization from PM to the ER for esterification there by the activity of cholesterol acetyltransferases ACAT1/SOAT1 and ACAT2/SOAT2. ER-PM contact sites and lipid transfer proteins are involved in non-vesicular transport of SM free cholesterol (Litvinov et al., 2018), and therefore, our CE detection in PM isolates most likely reflects co-purification of ER-plasma junctions. Total SM was not measurably reduced after TCR stimulation of CTRL cells, indicating that SM is still present to prevent removal of cholesterol and that this did not correlate with strong CE induction. Subbaiah and others (Subbaiah et al., 2008) demonstrated that rather ceramide generation and not sphingomyelin degradation is crucial for CE synthesis. Our lipid measurements showed a correlation of NSM2 dependent ceramide generation with the CE production after TCR stimulation.

Here we show general dysregulation of all steps involved in cholesterol homeostasis in NSM2 deficient Jurkat cells: CE production, cholesterol uptake, efflux and activation of cholesterol sensing transcription factor SREBP2, manifesting in increased cholesterol accumulation (Figure 4). NSM2 deficiency in *fro/fro* mouse resulted in storage of cholesterol in ear skin fibroblasts promoting lipid raft formation and activation of hyaluronan synthase (Qin et al., 2012). Similar, ΔNSM T cells showed high PM and intracellular cholesterol accumulation. Cholesterol homeostasis is fine-tuned by cholesterol uptake, efflux and regulation of transcription factors activating gene expression involved in lipid synthesis. Cholesterol uptake is mediated by serum lipoprotein LDL internalization by its receptor (LDLR) in clathrin-coated vesicles. Interestingly, exogenously added human urinary neutral sphingomyelinase can regulate LDL receptor activity, LDL uptake and cholesterol ester synthesis in fibroblasts (Chatterjee, 1993). Our data showed enhanced cholesterol uptake in NSM2 deficient Jurkat cells cultivated in serum containing medium (Figures 4B,C). However, expression of LDLR was not enhanced (data not shown). Surprisingly, we were able to reduce the cholesterol load in ΔNSM cells by supplementing them with C16-Ceramide, indicating that ceramides or possibly its metabolites balance cholesterol uptake by regulating membrane environment of uptake regulatory proteins probably by effecting lipid raft formation. As shown above, NSM2 regulates lysophospholipid levels and therefore possibly membrane curvature important for clathrin-coated vesicle formation involved in serum lipoprotein uptake. The exact mechanistic link between ceramides and LDLR mediated cholesterol uptake in T cells is not clear and stayed out of the scope of this study.

It has been shown that inhibition of ACAT1/SOAT1 or PKC regulates high density lipoprotein receptor (HDLR) dependent efflux of intracellular cholesterol (Mendez et al., 1991). Correlating with the inhibition of those pathways in NSM2-deficient cells, cholesterol efflux toward medium supplemented with recombinant HDL was significantly reduced (Figure 4D).

We observed increased cholesterol accumulation in NSM2-deficient Jurkat cells accompanied with less accumulation of cleaved active sterol regulatory element-binding protein SREBP2 which is more specialized in cholesterol regulation pathway (Figure 4E). When the sterol concentration in ER drops, SREBPs are transported by chaperone protein sterol sensing SREBP cleavage activating protein SCAP from the ER to the Golgi where SREBPs are cleaved by proteases. Cleaved N-termini of SREBP are released into the cytoplasm and can diffuse to the nucleus where they induce expression of target genes regulating biosynthesis and uptake of cholesterol (Litvinov et al., 2018). Negative regulation of SREBP in NSM2 deficient cells may result from enhanced delivery of the LDL-cholesterol, uptake of which is increased in NSM2-deficient cells (Figure 4B), to the sterol sensing ER compartment. Interestingly, NSM2-deficient cells could not activate SREBP2 even after cultivation in serum depleted medium, indicating that cholesterol removal from ER may be regulated by NSM2. That made them especially sensitive to the starvation induced cell death.

Now we show that NSM2 is a strong regulator of CE generation in Jurkat T cells. Yang and others proposed to use avasimibe, an inhibitor of the cholesterol esterification enzyme ACAT1/SOAT1, for therapy to improve CD8^+^ T cell mediated control of tumor growth (Yang et al., 2016). Their experiments were done in a mouse model and showed strongly enhanced anti-tumor activity of mouse CD8^+^ effector T cells treated with avasimibe or deficient for ACAT1. Now we show that *in vitro* TCR driven expansion of primary human T cells treated with avasimibe revealed no real improvement in the peripheral blood CD8^+^ T cell compartment, whereas CD4^+^ T cells were highly sensitive and showed significantly impaired proliferation (Figure 6). Accumulation of CE is associated with increased proliferation of tumor cells (Matsumoto et al., 2008). Also expansion of CEM and MOLT4 T cell lines is positively regulated by cholesterol esterification (Dessi et al., 1997). PKCζ/ERK signaling pathway is crucial in CE synthesis enhancing glioblastoma cell growth and invasion (Paillasse et al., 2009). Similarly ERK activation enhances CE synthesis in monocyte-derived macrophages (Napolitano et al., 2004). However, exact mechanism how CE effects cell proliferation is still poorly understood. Oxysterols, made from excess of free cholesterol accumulating upon CE synthesis inhibition, are supposed to play a negative role in cell proliferation and were suggested to use as a chemotherapeutic agents controlling tumor growth (Schroepfer, 2000). Here our data are suggesting that more detailed studies on properties of and differences between mice and human T cells and their subsets regarding CE function and cholesterol homeostasis are needed to understand the composite intervention of CE with tumor growth and tumor infiltrating T cells depending on availability of nutrients and type of tumor.

The results presented here indicate a tight connection between sphingolipid metabolism, TCR signaling regulated by NSM2 and cholesterol homeostasis in T cells, the deregulation of which can lead to metabolically orchestrated inflammation or negatively regulated T cell differentiation and anti-tumor activity. Immune cells sensitive to deregulated cholesterol metabolism and driving autoimmune diseases are poorly characterized. Studies of immune cell compartment specific NSM2 activity levels and responses to sterol metabolic stress would help to evaluate NSM2 significance in metabolically regulated inflammation.

## Data Availability Statement

All datasets generated for this study are included in the manuscript/Supplementary Files.

## Author Contributions

BK, LD, and EA: conceptualization. CB, BK, FS, and EA: methodology. CB and EA: formal analysis. CB, FS, and EA: experimental investigation. LD and EA: writing. BK: funding acquisition. EA: supervision.

## Conflict of Interest

The authors declare that the research was conducted in the absence of any commercial or financial relationships that could be construed as a potential conflict of interest.
