# Supplementary material for: Role of Neutral Sphingomyelinase-2 (NSM 2) in the Control of T Cell Plasma Membrane Lipid Composition and Cholesterol Homeostasis
Source: Front Cell Dev Biol. 2019 Oct 15;7:226. doi: 10.3389/fcell.2019.00226 (PMC6803391; doi:10.3389/fcell.2019.00226)
Supplement: Supplementary file 1 [file Table_1.DOCX]

**SFig.1**

**
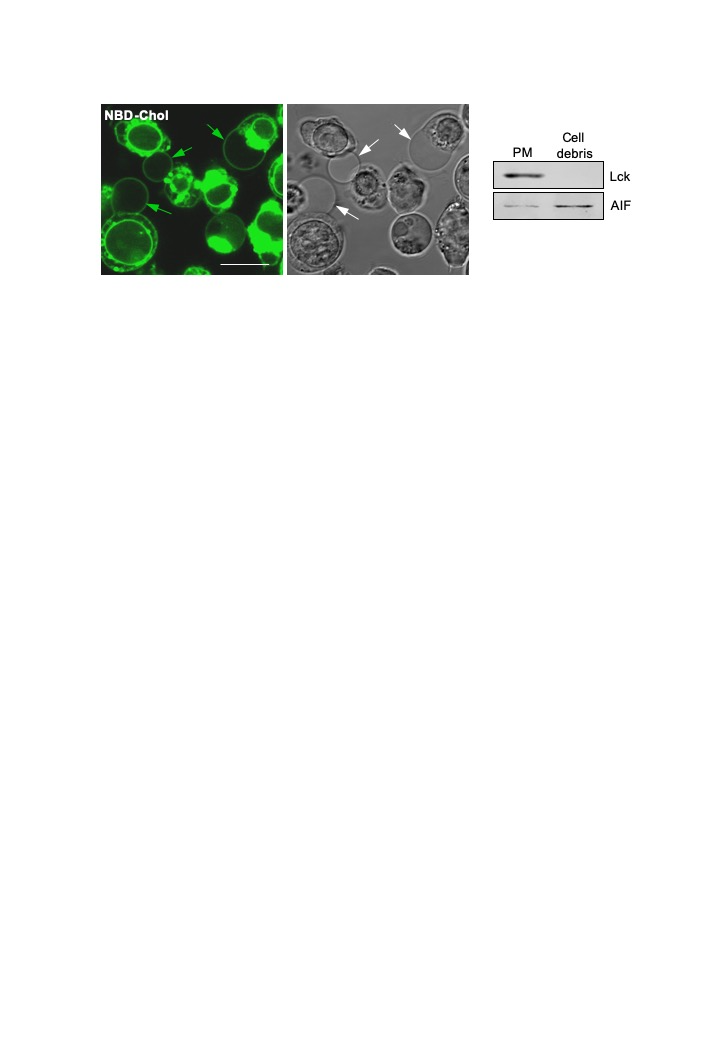
**

**SFig.2**

**SFig.3**

**SFig.4**

**SFig.5**

**
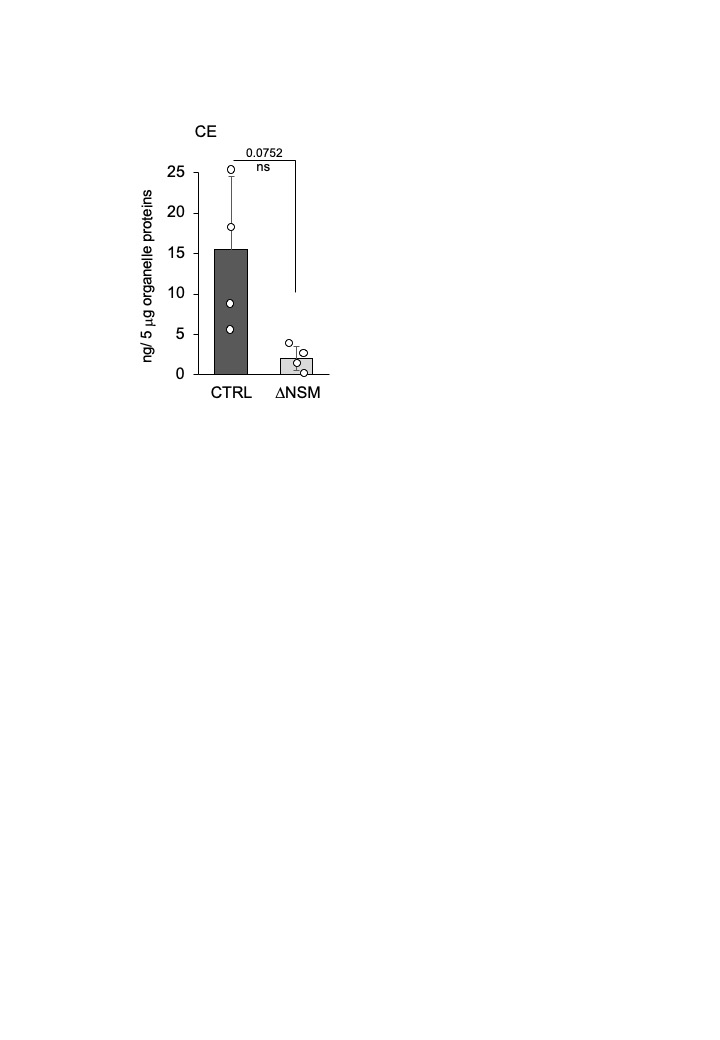
**

**SFig.6**

**Supplementary figures**

**Figure S1: Mitochondria specific protein present in plasma membranes isolated as giant PM vesicles (GPMVs)**

Cell associated and free-floating PM vesicles labelled by NBD-Cholesterol are shown in immunofluorescence picture (left panel) and in DIC picture (middle panel). Vesicles are marked with arrows. The cell compartment specific localization of Lck and AIF was detected in plasma membranes (PM) isolated as vesicles and cell debris of Jurkat cells by Western blot analysis (right panels). Scale bar: 10 μM.

**Figure S2: Plasma membrane localized ceramides are regulated NSM2 dependently**

LC-MS/MS (A, C) and direct infusion MS/MS (B) analysis of sphingolipids in isolated fractions of plasma membrane (A, B) or organelles (C) of CTRL and ΔNSM cells left unstimulated or α-CD3 stimulated for 10 minutes. Sphingolipid measurements shown in A and C were done at University of Potsdam. Cer in plasma membranes (A) were measured in one probe per cell type or stimulation and obtained values for ΔNSM cells were normalized against CTRL cells and combined with `Lipotype` Cer measurements to calculate Cer reduction shown in Figure 1C (left graph). Mean values with standard deviations of 3 (B) or 4 (C) independently performed fractionations are shown.

**Figure S3: NSM2 controls plasma membrane glycerophospholipids**

Direct infusion MS/MS analysis of phosphatidylcholin (PC) (A), cardiolipin (CL) (B), lyso-phosphatidylcholin (LPC) (C, left graph) and lyso-phosphatidylethanolamin (LPE) (C, right graph) content and species distribution in plasma membrane fractions of CTRL and ΔNSM cells left unstimulated or α-CD3 stimulated for 10 minutes. Mean values with standard deviations of the measurements of three independently performed fractionations are shown.

**Figure S4: NSM2 promotes TCR stimulation dependent upregulation of highly saturated diacylglycerol DAG species**

Direct infusion MS/MS analysis of DAG species was performed in plasma membrane fractions of CTRL and ΔNSM cells left unstimulated or α-CD3 stimulated for 10 minutes. Mean values with standard deviations of the measurements of three independently performed fractionations are shown.

**Figure S5: NSM2 promotes cholesteryl ester (CE) accumulation in intracellular organelle membranes**

CTRL and ΔNSM Jurkat cells were stimulated with α-CD3 for 10 minutes followed by organelle isolation, lipid extraction and CE detection by colorimetric analysis. Mean values with standard deviations of the measurements of independent cell extracts are shown. Each independent measurement is marked as a circle and p-value is shown.

**Figure S6: Inhibition of acyl-CoA: cholesterol transferases do not affect viability of primary human T cells**

Human CD3^+^ T cells were treated with ACAT1/SOAT1 specific inhibitor avasimibe (Ava) and ACAT2/SOAT2 specific inhibitor pyripyropene A (PPPA) in combination (10 μM each) for two days and cells positive for AnnexinV and propidium iodid (PI) were detected by flow cytometry.

**Excel file: Lipid analysis pmol.xlsx**

https://doi.org/10.6084/m9.figshare.9642905.v1
